# Urban Biodiversity, City-Dwellers and Conservation: How Does an Outdoor Activity Day Affect the Human-Nature Relationship?

**DOI:** 10.1371/journal.pone.0038642

**Published:** 2012-06-08

**Authors:** Assaf Shwartz, Alix Cosquer, Alexandre Jaillon, Armony Piron, Romain Julliard, Richard Raymond, Laurent Simon, Anne-Caroline Prévot-Julliard

**Affiliations:** 1 UMR 7533 CNRS, Univ. Paris 1-Panthéon-Sorbonne, Univ. Paris 7-Denis Diderot, Univ. Paris 8-Saint-Denis, Univ. Paris Ouest Nanterre La Défense, Lab Dynamiques sociales et recomposition des espaces (LADYSS), Paris, France; 2 UMR7204, CNRS-MNHN-UPMC, Lab Conservation des Espèces, Restauration et Suivi des Populations (CERSP), Muséum National d'Histoire Naturelle, CP 51, Paris, France; 3 Institut des Sciences Humaines et Sociales, Laboratoire d'Anthropologie Sociale et Culturelle, Université de Liège, Belgium; 4 Institut des Sciences de la Communication du CNRS (ISCC), Paris, France; University of Western Ontario, Canada

## Abstract

Urban conservation education programs aim to increase knowledge and awareness towards biodiversity and to change attitudes and behaviour towards the environment. However, to date, few urban conservation education studies have evaluated to what extent these programs have managed to achieve their goals. In this study, we experimentally explored the influence of an urban conservation activity day on individual knowledge, awareness and actions towards biodiversity, in both the short and longer term.

We organised three activity days in Paris (France), during which people were invited to participate in urban conservation efforts. Both quantitative (questionnaire) and qualitative (interviews) methods were employed to investigate the influence of this short urban nature experience on the relationships that city-dwellers develop with nearby biodiversity. We found a strong positive correlation between the levels of participation and an immediate interest towards local urban biodiversity. In the longer term, however, although participants claimed to have gained more knowledge, local awareness and interest for species in their daily environment, they did not seem to extend this interest to participating in other related activities. These results highlight the complexity of validating the effectiveness of this type of education program for achieving conservation goals. Although such a short activity may only have a limited environmental impact, it nevertheless seems to increase people's knowledge, awareness, interest and concern. We therefore believe that when repeated locally, these short conservation education programs could enhance people's experience with nature in cities and achieve conservation goals more fully.

## Introduction

Sprawling urban landscapes are a growing threat to biodiversity conservation [1] and are assumed to separate the majority of the world's population from the biological reality and functions of the natural world [2], [3]. However, green areas in cities can harbour wildlife and can even host a rich diversity of species [4]. Urban nature (here broadly referred to as the non-human world) thus offers a dual ecological and social challenge: (1) to preserve urban biodiversity to contribute to global conservation [5] and (2) to increase interactions between city-dwellers and urban fauna and flora to reconnect people with nature [2], particularly through educational programs [6].

Conservation education is a part of environmental education, i.e., a “learning process that increases people's knowledge and awareness about the environment and associated challenges, develops the necessary skills and expertise to address these challenges, and fosters attitudes, motivations, and commitment to make informed decisions and take responsible action” [7]. In this sense, conservation education aims to increase awareness and knowledge and to change attitudes towards biodiversity conservation among general audiences, scientists and policymakers [8], [9]. In the last two decades, interest in conservation education has increased [10] and various programs have been promoted worldwide in natural history museums, zoos, botanical gardens, natural or semi-natural parks and reserves [11]. These programs target different segments of the public (e.g., children, teachers, farmers, and managers), who may have different types of relationships with the environment (e.g., visitors of nature reserves *versus* residents) [9], [12]. Finally, these programs use a variety of methods, from presentations of conservation issues, concerns, and practices (e.g., [11], [13]) to more integrated programs designed to enable people to participate in conservation research, decision-making, and action (e.g., [8], [14], [15]).

Some conservation education programs are still based on the knowledge deficit model, which assumes that people do not become involved in a given issue because they lack knowledge (e.g., [16], [17]). However, in addition to this top-down knowledge transmission pattern [18], many initiatives now combine outdoor activities with emotional, aesthetic, and creative involvement, which have positive effects on individual awareness of biodiversity (e.g., [19], [20], [21]). These action-based programs may achieve better results in influencing pro-environmental behaviour [22], [23], [24]. Education programs that combine information transfer with an empirical approach are assumed to have more value for biodiversity conservation [25]. Indeed, these activities may be more prone to combining cognitive, affective and behavioural components of environmental education [26].

Several studies have assessed whether these different approaches can have a conservation impact [27], [28]. However, the empirical difficulty in measuring changes in attitudes after taking part in a conservation education program and in quantifying any conservation impact may make it difficult to achieve unambiguous results [14]. Studies have revealed positive changes in attitudes and intentions towards conservation in the short term, just after taking part in the program [29]. However, these changes often wane within a few months after the activity, although a traceable impact may remain [30],[31]. Other studies have found that the duration of the education program was a significant predictor of its effectiveness (reviewed by [32]). However, most of this knowledge was gathered from children and adolescents [28]. The influence of conservation education programs on adults' attitudes and practices is relatively unstudied. However, addressing this value-action gap between individual knowledge, declared intentions and subsequent behaviour is one of the keys to monitoring effective environmental protection and biodiversity conservation [26], [33], [34].

Although several authors have already stated the potential importance of urban biodiversity for conservation education [2], few studies have shed any light on the effectiveness of these programs in the urban environment. Two participatory conservation programs [8], [15] and one top-down outdoor activity for children [35] have shown an increase in knowledge and pro-conservation attitudes among participants immediately after the activity. In the urban context, however, we are not aware of any study that has investigated this increase in the longer term or the effect of the pro-environmental profiles of the participants (i.e., accounting for the fact that the participants in conservation activities may already have a pro-environmental profile, as mentioned by [8], [15]).

Here, we used an interdisciplinary approach to study the influence of an empirical urban conservation education program on individual interest for urban fauna and flora (what we refer to as biodiversity) both in the short term (immediately after the program) and in the longer term (a few months later). We aimed to explore how partaking in a short empirical semi-participative (i.e., knowledge-based education and participation in conservation effort activities) urban conservation activity day can influence individual interest in local urban biodiversity. For each participant (and accompanied children), we followed their level of involvement in the activities and investigated whether greater involvement was associated with a higher short-term interest in additional urban nature activities. In parallel, we identified the profile of each adult participant (using a questionnaire) to assess the degree of environmental concern of each participant. Finally, we used qualitative methods (phone and face-to-face interviews) to explore changes in knowledge and pro-environmental attitudes in the medium term, especially concerning local biodiversity.

## Methods

### Organisation of the activity day

We organised three identical conservation activity days for the general public in three different small gardens (approximately 1 ha) in residential areas of Paris, France. These activity days were part of a larger research project conducted since 2009 that explores the means to conserve or increase biological diversity in the small gardens of Paris. The main aims of these empirical semi-participative activity days were twofold: (1) to expose residents to the urban biodiversity and particularly the species, i.e., birds, spontaneous plants (“garden weeds”) and pollinating insects, that can be found nearby; and (2) to give residents an opportunity to actively get involved in a project intended to increase local biodiversity.

To contact the residents who live in close proximity to and regularly visit the gardens, we advertised the events in a 500-metre radius neighbourhood around each garden. We distributed 4500 flyers in mailboxes in the streets surrounding the gardens and put up posters in public places (e.g., gardens, bakeries, and grocery shops). The municipality of Paris also announced these three activity days on its website and in its monthly events magazine. The activity days were conducted during school holidays, on 27–29th April 2010, and were designed to be attractive to both children and adults. Five different activities were repeatedly proposed throughout the day by a team of researchers from the National Museum of Natural History (Table 1). The first two activities invited participants to take part in local conservation efforts. The other three activities allowed participants (children and adults) to explore and interact with the diversity of species and to learn about their biology and related conservation efforts (Table 1). Additionally, a small poster exhibition showed the diversity of birds, spontaneous plants and pollinators sampled in each garden in 2009 and the diversity of the different taxonomic groups in the urban environment.

**Table 1:** Description of the five activities proposed to the participants during the three activity days.

Activity name	Description	Aim	Participants
1. Gardening	Helping gardeners to sow and plant a 30-m^2^ flower meadow to attract a variety of pollinators.	Participative (Children & adults)	20
2. Hotel for pollinators	Building boxes for pollinators in wood and bamboo (*Bambusoideae*) to provide nesting opportunities for different pollinating insects. Participants could build small boxes to take home or place in the garden or help to build large pollinator “hotels”.	Participative (Children & adults)	31
3. Nesting birds	Discovering the bird species that nest in the gardens and learning to recognise birds by their song.	Knowledge (Children & adults)	50
4. Treasure hunt for spontaneous plants	Participants were given a map of the garden and pictures of eight spontaneous plant species, which they then went looking for. The activity ended with explanations on the species found.	Knowledge (Children)	23
5. Miniature garden	Creating a small garden in a sandbox using only natural materials (e.g., tree bark, leaves, pebbles, mosses).	Interactive (Children)	42

The aim of each activity is classified into three categories: (1) learn about urban nature (“knowledge”); (2) participate in conservation efforts (“participative”) (3) interact with natural features (“interactive”). The activities were held throughout the day; both adults and children participated, but only the adults were followed and registered (total number of registered participants: 102).

Flyers on eleven free-of-charge activities on urban nature that were to take place in the following months were distributed in each garden (Table 2). These flyers offered participants opportunities to widen their interaction with urban biodiversity both locally (i.e., in the same garden or at home) or in the Paris area. We selected and designed these advanced activities to be attractive to various types of people, including parents and children. The flyers were made by the authors to ensure that they were as similar as possible in the design, colour and pictures used. The flyers were laid out on a table a short distance from the main activities. After each activity, people were informed about these flyers without being taken to the table. We made the assumption that the quantity of flyers taken reflected (and was a proxy of) people's short-term interest in widening their interactions with urban biodiversity.

**Table 2:** Description of the eleven further activities proposed to participants by the during the activity days.

Activity name	Description	Location	Interested
Bird watching	During weekends in May, observing and listening to the birds in the garden.	Local	22
Placing nest boxes	Participants were invited to come one afternoon in early June to help place the pollinator hotel and the small nests they built themselves.	Local	20
Pollinator watching	Observation of a flower meadow and the pollinators visiting it in July.	Local	17
Garden butterflies	Offer to take part in the garden butterflies watch program.	Local	15
Pollinator friendly planters	Instructions on how to create a pollinator-friendly planter on a window ledge or balcony.	Local	17
Urban nature walk	A short urban nature walk around the green belt of Paris.	Paris area	14
Botanical garden	Invitation to visit a “floral park” in Paris (with general information provided).	Paris area	15
Plant fair	An opportunity to exchange plants and gardening materials for free.	Paris area	13
Discovering amphibians	Information on an activity day on amphibians during a nature festival organised by the National Museum of Natural History in Paris (May).	Paris area	14
Gardening	Information on how one can do shared-communal gardening in the city of Paris.	Paris area	10
General information	Information on several biodiversity activities organised by the city of Paris in the summer and spring 2010.	Paris area	8

The location of each activity is classified into local, i.e., in the same garden or at home, and Parisian urban area, i.e., activities that took place in the Paris metropolis.

### Data collection

We followed the participants during the activity day to assess their participation level and short-term interest for the further activities. We registered each adult participant (providing a ticket with a reference number) at the entrance, and we noted whether he/she came with children. To explore the relationship between short-term interest (number of flyers taken) and the participation level (i.e., number of activities each adult and accompanied children take part in; item *number of activities*), we recorded each person's reference number at the beginning of each activity and when they took flyers.

At the end of their participation, we asked people to fill in a short questionnaire to characterise their social and pro-environmental profile as well as garden-related information (translated version in Text S1). The social profile was assessed from information on gender, age, marital status, income perception (0–10 scale, item *income*) and education level (item *qualification*). The pro-environmental profile was assessed from childhood settlement type (1 [big city] – 5 [farm], item *Childhood*), holiday choices (open question, answers later classified into three categories: no relation to nature, open areas and nature; item *holidays*), and whether they had plants (item *plants*) or pets (item *pets*) at home. Garden-related questions included whether the participants had been informed about the event, how frequently (item *frequency*) and why they visited the garden (an open question). Two relevant reasons to visit the garden were then classified into two separate binary variables based on whether people mentioned that they visited the garden to interact with nature or for children's recreation (items *interact with nature; children's recreation*). Similar questions had already been asked in the same gardens as part of the larger research project (n = 408 people) between March and August 2010 (see Text S2 for further details on this survey). This step enabled us to compare our results with the profiles of people who visit the gardens on a regular basis (referred to here as “general visitors”).

We later contacted participants who agreed (n = 56) to share their experience during the activity day to find out whether they participated in any of the further activities. In August and September 2010 (i.e., 4–5 months after the activity day), we managed to establish phone contact with 26 participants with whom we briefly explored whether they had participated in or implemented any of the additional activities suggested. Among these 26 participants, only 13 agreed to meet for a face-to-face semi-directive interview (see Text S3 for the interview guidelines and Table S1 for the profiles of the interviewees). The aim of these interviews was to learn more about the interviewees' motivations for participating in the activity days, their assessment of the activity day and the potential impacts produced by their participation.

We recorded the number of people that came to each of the three local activities organised in each garden during the spring and summer (Table 2). We used this information, together with the phone and face-to-face interviews, as a way to explore whether people participated in further activities in the longer term (1–3 months).

### Data analyses

We explored the differences between the profiles of participants and general visitors using a general linear model with a binomial error structure. All of the social, pro-environmental profiles and garden variables were entered into the model (Table 3). We then explored the relation between the number of flyers taken (dependent variable) and the participation level (*number of activities*), while controlling for relevant profile and garden variables (i.e., variables we expected to influence the number of flyers taken; Table 4). We used a generalised linear model with a quasi-Poisson error structure. All linear models were fitted using a stepwise backwards procedure followed by a forward procedure [36] until only significant terms (P≤0.05) remained in each model. We tested for the absence of significant collinearity between the independent variables and for each model's assumptions using residual and leverage plots [36]. All of the statistical analyses were performed in R.2.12.2 (R Development Core Team 2007). We transcribed and analysed the semi-directive interviews through a qualitative ground-theory approach [37].

**Table 3:** Results of the general linear model with binomial error structure comparing differences between the social and pro-environmental profiles of people who came to the activity (participants; n = 69) to people who visit the gardens on a day-to-day basis (general visitors; n = 408).

Variables	Estimate ± S.E	Df	P-value
Intercept	−4.20±0.71	1	<0.001
Gender (Male)	−1.08±0.36	1	0.002
Age	0.03±0.01	1	0.003
Marital status (Single)	−0.38±0.32	1	0.241
Income	0.03±0.09	1	0.728
Qualifications	−0.02±0.07	1	0.736
Childhood (City)		4	0.965
Childhood (Town)	0.07±0.40		
Childhood (Small town)	−0.12±0.42		
Childhood (Village)	−0.10±0.44		
Childhood (Farm)	0.02±0.82		
Holidays (No relation to nature)		2	0.011
Holidays (open-air)	1.25±0.46		
Holidays (In natural environment)	0.60±0.48		
Plants at home (Yes)	0.68±0.42	1	0.107
Interact with nature (Yes)	1.28±0.4	1	0.001
Children's recreation (Yes)	0.83±0.31	1	0.008
Frequency of garden visits	0.01±0.01	1	0.508

Adjusted effect size±standard errors, degrees of freedom and p-value for minimal models (all significant term included), whereas coefficients and p-values of non-significant terms are obtain by fitting each term separately into the minimal model.

**Table 4:** The results of the general linear model, with quasi-Poisson distribution errors, are given to account for the variance in taking flyers, by profiles, garden variables and the number of activities in which each attendee participated (n = 69).

Variables	Estimate ± S.E	Df	P-value
Intercept	0.26±0.56	1	0.639
Gender (Male)	−0.36±0.57	1	0.466
Age	0.02±0.01	1	0.015
Income	−0.36±0.09	1	<0.001
Qualifications	−0.02±0.09	1	0.818
Childhood (City)		4	0.074
Childhood (Town)	−1.06±0.47		
Childhood (Small town)	−1.21±0.63		
Childhood (Village)	−0.32±0.58		
Childhood (Farm)	−0.60±0.73		
Holidays (No relation to nature)		2	0.846
Holidays (open-air)	−0.15±0.44		
Holidays (In natural environment)	−0.26±0.46		
Plants at home (Yes)	1.24±0.76	1	0.108
Pets at home (Yes)	0.67±0.29	1	0.027
Interact with nature (Yes)	−0.06±0.35	1	0.870
Children's recreation (Yes)	−0.16±0.37	1	0.661
Number of activities attended	0.34±0.08	1	<0.001

For non-significant variables, coefficients±SE and p-values are presented at the step of exclusion from the model. Adjusted effect size±standard errors, degrees of freedom and p-values for minimal models (all significant terms included), whereas coefficients and p-values of non-significant terms are obtain by fitting each term separately into the minimal model.

### Ethics

Our research activities fall within the scope of categories exempt from IRB approval. The entire process of participation was strictly anonymous and people were assigned numbers. All participants were city-dwellers, and the participation was entirely free of charge and engagement. Participants were informed verbally of the broad aims of the research and chose whether they wanted to answer the questionnaire. Among those who answered the questionnaire, several agreed to provide their contact details (i.e., mail or telephone) to be contacted later to give their opinion about the activity days; this was encouraged, but not compulsory. All children who participated in the event were accompanied by a parent/adult referent. Children were not monitored for the study (questionnaire and interview).

## Results

More than 250 people took part in the three activity days, the majority being children. We monitored the participation of 102 adults, who came with approximately 128 children, but the children were not considered in the analysis of this study. Most of the 69 people that agreed to answer the questionnaire at the end of the activities (69%) knew about our activity day and had deliberately come to take part in it. Others had come to visit the garden, where they discovered the activity day and chose to participate. Most adult participants (80%) lived close to the gardens (i.e., within a 500-metre radius). Subsequent detailed interviews (n = 13) suggested that the main reason for coming was curiosity, both for the theme (biodiversity-related activities) and the location (in their neighbourhood): *“Curiosity… To find out…Because we've got the impression that there are no insects, no animals here…”* (*7*; number indicates the interviewee's profile; Table S3). The activities were perceived as an opportunity to acquire or deepen knowledge about the daily environment: *“I'm unemployed now, so I have free time, I live in the neighbourhood and I often come to this garden […] so I came to this event to find out more about the area” (6).*

### Social and pro-environmental profiles

The social and pro-environmental profiles of the 69 adult participants who filled out the questionnaire were somewhat different from those of the general visitors (n = 408): most of the adult participants were women (81%), a significantly higher proportion than for general visitors (64%; Table 3). The average age of participants (50 years old) was significantly higher than the average age of general visitors (44 years old; Table 3). Both participants and general visitors said they visited the garden frequently (14.6 vs. 14.2 visits per month). However, participants differed significantly from general visitors in their reasons for those visits (Table 3): while 7.6% and 49% of the general visitors come to the garden to interact with nature and for children's recreation, respectively, the corresponding proportions were 27.5% and 62% for participants. Finally, the proportion of people spending their holidays in the open air or in nature was significantly higher among the participants than among general visitors (Table 3).

In the longer term, the 13 interviewees did not mention any specific environmental interest or actions before the activity day. Most of the interviewees (n = 11) had no previous similar experience (i.e., biodiversity discovery) in an ordinary context. Nevertheless, six people said they visited nature museums and environmental exhibitions. However, most of their motivations were not consciously related to the environment but more to recreation, social encounters, entertainment and children's education. Five interviewed people did have previous knowledge and experience of local urban biodiversity and were sensitive to their everyday life environment: “*In the morning, I hear birds […] they start singing at 5 a.m., “tatintintintatatintin”, and they gossip, they tell a lot of stories*” (7). These relationships led to the development of empirical knowledge: “*before I had bees coming to my balcony all summer long […] Now, I don't have bees anymore, only wasps and bumblebees*” (11).

Qualitative interviews also suggested that the ordinary local urban setting is the actual reference for people's perceptions of nature but that these perceptions are sometimes negative. Non-urban nature and urban nature were strongly opposed, the first being considered as “real” nature, the second as a poor substitute: “[*Urban*] *biodiversity is limited, and I don't think it's healthy biodiversity. Because pigeons carry germs […] I don't think we have a good ecology in Paris, well we try, but it's nothing like in a provincial city*” (10). Approximately one-third of the interviewees (n = 5) raised the issues of conservation and environmental action. However, the interviewees described themselves as not very active and confused about the type of action they could undertake: “*Biodiversity is about so many things, we've been hearing about it for a few years now on TV, but people don't really know any more about it. […] I have a lot of things in my head, but I find it hard to take any action*.” (5)

### Activities during the events

The quantitative results from the 69 questionnaires showed that the most attractive activities were those involving birds because they attracted children, their parents and older people who did not accompany children (Table 1). The two children-dedicated activities and the building of nest-boxes for pollinators attracted mostly children with their parents (Table 1). Only a few people participated in the gardening activity, which took place only three times during the day (due to the limited surface that needed planting). Further interviews demonstrated that people were very happy with the program we offered for various reasons, including allowing children to touch natural elements (miniature gardens): *“they touched the sand, they touched the ground, the leaves… That's great, it's direct contact. Parents so often say “don't touch that!”* (13) and allowing knowledge acquisition from ecologists: “*We really learned things from him. […] It was interactive; it was fun as well, because he brought some recordings to listen to birdsongs, to communicate*.”

### Interest in urban nature-related activities in the short and long term

Of the 102 registered participants, 43% (n = 44) took flyers. Flyers advertising local activities (Table 2) were taken significantly more often than Paris area flyers (Mann-Whitney Z = 2.66, p = 0.004). “Flyer taking” was positively correlated with age and pet ownership and negatively with salary levels (Table 4). However, pro-environmental profile variables such as visiting the garden to interact with nature and holidays did not have a significant effect on flyer taking (Table 4). The number of flyers taken was significantly positively correlated with the number of activities people took part in during the activity day (Table 4).

In the longer term, we did not record any engagement in the further urban nature activities we proposed in the gardens and in the Paris area. Apart from three children, nobody came to any of the three further activities we offered in each garden. None of the 26 people who we interviewed by phone stated that they participated in any of the proposed activities. However, among the 13 interviewees, five said they were interested in participating in similar activity days and even suggested further activities based on a local, regular and tactile approach.

However, some people (n = 4) installed insect nesting boxes on their balconies and checked on the results: “[*The grandchild] installed it in his home […]. He told me that no insects came*.” (2). For one participant, taking part in the activity day had encouraged an engagement with biodiversity through new practices: “*It encouraged me to take part in “nature” activities. […] We built little walls, we made shelters for hedgehogs! You know, it really motivated us…*” (8). The interviews also revealed substantial changes in people's consideration of their nearby environment. Seven people discovered species present in the garden: “*I had no idea of what you made me discover. We walk through [the garden] and then we see flowers, and we don't see anything, anything at all”* (11); *“I never imagined there were so many birds in Violet square.”* (13). This new awareness may have led to introspection and reflection on the partial view of the biodiversity that people encounter on a daily basis: “*We go to the park, we do almost the same thing every time, and it's true, we don't necessarily realise all these things going on around us*” (12). The insect nesting boxes installed at home gave people an opportunity to make regular observations and discover how biodiversity functions, creating an immediate link between a daily practice and biodiversity conservation: “*I saw bees, they buzz around, searching; I have flowers on my balcony, so they come. There are holes of some kind under my plastic chairs, and they try to go in there and not in the nesting box I put up*” (7). “*Insects too, what interested me very much was that I didn't know we could help them to settle*” (11).

## Discussion

In this study, we explored the consequences of taking part in a short semi-participative activity day on urban conservation. The research focused on people's interest and awareness in local urban nature and on their concern for conservation. Our results demonstrated that local environmental activities could be attractive to urban dwellers and especially to parents of young children. Our quantitative and qualitative results showed that participating in biodiversity activities could increase people's knowledge, awareness and interest in urban biodiversity in the short term. These findings are partly consistent with other studies on education in non-urban environments [9], [14], [28]. These studies have also demonstrated an increase in knowledge, concern and behavioural intentions immediately after participating. However, we found that this increase did not encourage individuals to further participate in biodiversity-dedicated activities.

Most of the participants were women (mothers or grandmothers) who visited the gardens with/for their children (77% of adult participants) and lived nearby. Most participants were children-minded and therefore had competing interests due to constraints related to child rearing. This could explain the lack of participation in the further activities (note, however, that eight out of the eleven further activities were directed at families). As families represent approximately 40% of the Paris population [38] and 49% of the gardens' general visitors, it seemed highly relevant that we understand their interactions with urban biodiversity and their response to conservation education programs.

The participants also seemed to be more environmentally sensitive than general garden visitors, as they preferred to spend their holiday in the open air and sought interaction with nature when visiting the gardens (Table 3). As in other studies [15], [35], these results could have confirmed that conservation education programs tend to attract an environmentally sensitised audience. However, our qualitative results showed that people's motivations for attending the activity day were less related to an existing interest in nature than with the proximity of the event and their own curiosity (raised by local advertising). We therefore suspect that in their answers to those two questions, the participants might have been influenced by the topic of the day and accordingly adapted their answers, especially because those questions were open-ended. The results of the qualitative interviews provided additional support to this hypothesis, as all interviewees mentioned a lack of knowledge of local urban biodiversity. These people, who are characterised by limited environmental concern, a lack of knowledge and mostly children-rearing interests, may also have limited experience with nature. Thus, they constitute a valuable group for conservation education programs [2], [9]. Our results indicate that the short type of activity we offered attracted this group of people and raised short-term and local interest, but it did not impact participation in further nature actives.

We considered “flyer taking” as a proxy for a short-term interest in urban biodiversity, and actual participation in the further activities was proposed as a sign of longer term interest. The flyer-taking proxy could have potential flaws, as the flyers' aesthetics and people's concern for the environment (e.g., paper consumption) could have influenced the number of flyers taken. For that reason, both the table's location and the flyers' design were planned carefully to provide a modest level of attractiveness and thus avoid any habitual flyer taking. Indeed, only 44 participants approached the table and took flyers, whereas the majority of adult participants (n = 62) were indifferent to the table, and only three participants took all flyers available. Most of these 44 people appeared to read and compare flyers, showing an interest in the further activities. Moreover, as we discussed above, our impression was that the participants did not demonstrate strong environmental concern. Therefore, although we cannot overrule this hypothesis, we believe that concern for paper consumption or attractiveness of flyers did not bias the results. Additionally, the proposed activities were all free of charge, avoiding a potential money-related bias in the choices [39]. We therefore believe that flyer-taking served as good a proxy for immediate interest. Evidence for an increased interest in urban biodiversity and biodiversity knowledge was also recorded in the 13 face-to-face interviews in which seven of the interviewees specifically mentioned that the activity day stimulated their curiosity for urban nature: *“It is true that I never though about taking interest in Violet's square fauna”* (13).

Our finding that immediate interest toward conservation was not translated to further actions in the longer term (which is often a challenge in conservation education [9], [14]) coincides with results of other studies in the field of psychology [40] and with assessments of other conservation education programs [9], [14], [28]. The strong correlation between the level of participation and interest (i.e., number of flyers taken) may suggest that interest in nature activities was increased during the activity day. However, our qualitative results in the longer term showed that although people gained knowledge and curiosity for their local biodiversity, they did not actually seem to engage in further biodiversity-dedicated activities. This result could highlight a gap between intentions in the short term and interest in longer term, but it could also be explained by the nature of the participants, who may have time constraints related to child-rearing.

The face-to-face interviews gave us an opportunity to investigate this gap and to explore the influence that this type of activity day can have on participant's awareness of urban biodiversity. During the activity days, people acquired knowledge on local biodiversity and discovered that they shared a common environment with wildlife. The interviews showed that this increased awareness of local species may have made people reconsider their local urban environment, which is consistent with the fact that the most attractive flyers were those advertising activities taking place locally (Table 2). This result is also consistent with other studies that showed the importance of close contact with ordinary local biodiversity [41] to increase people's interest in conservation in general [19], [42]. In cities, Evans *et al.* (2005) demonstrated that knowledge of urban biodiversity can be related to developing awareness and concern for urban nature. This awareness could be the first step in subsequent decision-making regarding conservation activities [41], [43].

Following the theory of planned behaviour [44], [45], we postulate that a shift in perceptions is a prerequisite for action. Our interviewees said that their non-participation was mostly due to a lack of time and flexibility and not due to lack of interest. We found that nature-related beliefs and activities appeared to be in competition with many other beliefs and activities that invite people's responses in their everyday lives [43], [46]. Although most of the further suggested activities were local, they required people to change their daily routine to deliberately participate in the activity. The hypothesis that the regular implementation of biodiversity observation in the individuals' routine is important was supported by the fact that people implemented some conservation actions at home and in a private context (nest-boxes, pollinator-friendly planters). Although they did not come back to the gardens on the suggested dates, several interviewed people showed enthusiasm for similar activity days. Thus, we believe that implementing activity days on a regular basis and in accordance with individuals' everyday routines involving children's recreation for instance could encourage people to prioritise their choices and to introduce biodiversity care in their daily lives. A single activity day was already sufficient to produce changes in individual knowledge and awareness of urban biodiversity for some people.

We suggest here three important features that can improve the efficiency of nature-related activities aiming at increasing individual awareness. First, being local appears to be a key factor for involving people. Second, the activities should aim to give local residents a central role through activities combining elements such as science, personal observations, games, and emotions [14], [24], [47]. Finally, we suggest that to increase the efficiency of conservation education, it is important to develop long-lasting programs that integrate observations and interaction with nature as closely as possible into people's daily lives [41], [48].

## Supporting Information

Text S1 **The questionnaire identifying social and pro-environmental profiles and garden-related information was presented to adult participants during the activity days.**
(DOCX)

Text S2 **Methods for the general survey in the gardens that was done independently from the activity days and allows comparing participants to general visitors.**
(DOC)

Text S3 **The interview guidelines followed (set of themes and questions used to frame the interview).** Face-to-face interviews were conducted, three and a half months after the activity days.
(DOCX)

Table S1 **Description of the social and pro-environmental profiles of the participants interviewed a few months after the activity days.**
(XLSX)
