# Supplementary material for: Urban Biodiversity, City-Dwellers and Conservation: How Does an Outdoor Activity Day Affect the Human-Nature Relationship?
Source: PLoS One. 2012 Jun 8;7(6):e38642. doi: 10.1371/journal.pone.0038642 (PMC3371046; doi:10.1371/journal.pone.0038642)
Supplement: Text S1 — The questionnaire identifying social and pro-environmental profiles and garden-related information was presented to adult participants during the activity days. (DOCX) [file pone.0038642.s001.docx]

**ID : _______________**


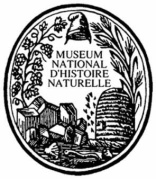


***Intro :*** *Good morning, I work as a researcher in the Natural History Museum (which organizes this activity day in the garden).In the context of our researches, we are investigating people’s participation to the activity days. The outcome will help us to improve their organization. This questionnaire is anonymous and will last less than five minutes. Will you agree to answer to these questions?*

**Questionnaire :**

| 1. **Did you participate to this activity day?** No / Yes**🡪 To which activities?**1, 2, 3, 4, 5, 6 |
| --- |
| 1. **How did you learn about this activity day?** Leaflet / Poster /Agenda of Paris city Hall / Website of Paris city **/** Word of mouth / Not informed /other :________________________________ |
| 1. **Do you have plants at home?** Yes / No    - **Where ?** Indoor**/**On the window side/Balcony**/**Terrasse / Indoor yard**/**Little garden/Garden/parcel of land |
| 1. **Do you have pets?** Dog / Cat / other(s): _____________________________________ |
| 1. **During summer, how many times per month do you come to a public garden?** ___________________________    - **What do you like to do in the garden?**_______________________________________________________ |
| 1. **Where did you spend most of your childhood (between 4 and 16 years old) ?** large city / mid-size city / small city / village / hamlet |
| 1. **This summer, what would you like to do during your holidays?** _______________________________________ |
| 1. **How do you perceive your home income?**  \| Low Average High \| \| \| \| \| \| \| \| \| \| \| --- \| --- \| --- \| --- \| --- \| --- \| --- \| --- \| --- \| --- \| \| 1 \| 2 \| 3 \| 4 \| 5 \| 6 \| 7 \| 8 \| 9 \| 10 \| |
| 1. **Are you a member of one [or more] association(s) or federation(s)?** Yes / No  - Which one(s) : _____________________________________________________________________ |
| 1. **Do you read one [or more] specialist magazine(s)?** Yes / No   Which one(s) : _________________________________________________________________________ |
| 1. **Gender : M / F. Your birth year ?_________________ Status**: single / couple   **Do you have children/grandchildren** (less than 15 years old)? _________________________________________ |
| 1. **What is your last diploma? _**_______________________________________________________ |
| 1. **Do you live in the neighborhood?** Yes / No. Could you indicate the name of your street?__________________and a n interval of numbers (for the large streets)? _________**____** |
| **“Thank you very much for your participation! We will continue our research on the garden during the coming months. Will you agree to discuss again with us about nature, in june, in order to deepen this study?”**  **🡺 If yes :your details ?**  Phone :____________________________  E-mail :________________________________________ |
|  |
| Comments :  (« You may leave us here your questions or observations, as well as complementary information ») |
