# Supplementary material for: Urban Biodiversity, City-Dwellers and Conservation: How Does an Outdoor Activity Day Affect the Human-Nature Relationship?
Source: PLoS One. 2012 Jun 8;7(6):e38642. doi: 10.1371/journal.pone.0038642 (PMC3371046; doi:10.1371/journal.pone.0038642)
Supplement: Text S2 — Methods for the general survey in the gardens that was done independently from the activity days and allows comparing participants to general visitors. (DOC) [file pone.0038642.s002.doc]

*Surveying general visitors to the gardens*

As part of larger project we interviewed 408 garden users filling up a questionnaire, after verifying that interviewees were frequent visitors to each garden. We developed a survey consisting of 34 questions that addressed a range of garden, well-being and biodiversity related topics, questions indentifying interviewees’ socioeconomic and environmental profile (see below). In order to compare general visitors to the people who participated in our activity we only use garden related socioeconomic and environmental profile question (questions 1,2,23:32) and compare them to the results from the questionnaire we used during the activity days. People were interviewed in both weekdays and weekends between March and August (2010). The people who passed the questionnaire in the larger project did the same job during the activity-days. Questions defining pro-environmental and socio-economical profiles in this questionnaire (below) and in the one that was passed during the activity day were identical except the additional question about pets at homes, journal subscription and association membership. To date, this data has not been published elsewhere.


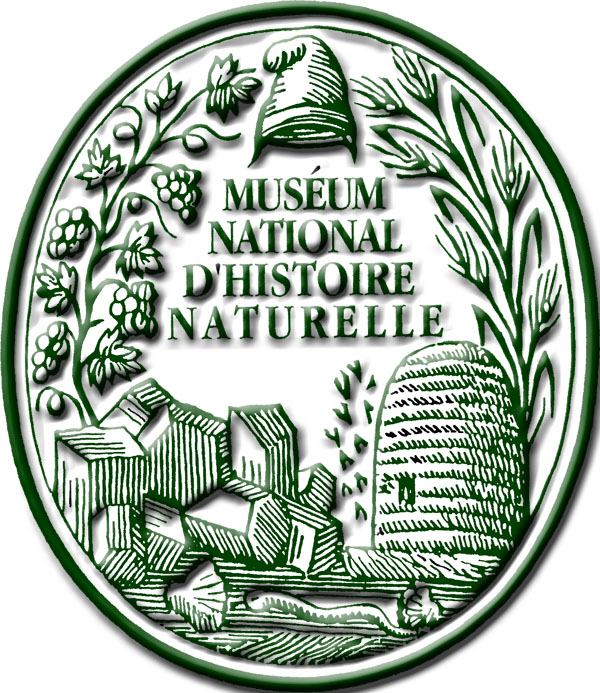

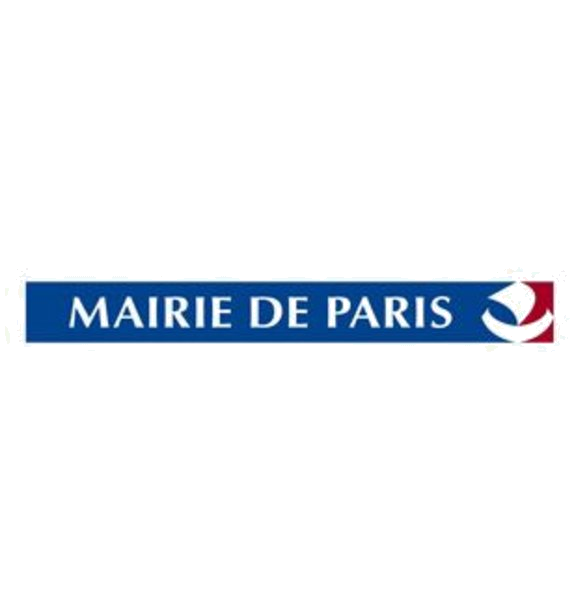
**Questionnaire**

Is this the first time you visit this garden? Yes / No

If not = 1. How many times a month you visit this garden? _______________________________
 2. What do you like to do in the garden? __________________________________________

*All the questions in this questionnaire are anonymous and will be used only in sake of this research. There are no good or bad answers; we are simply interested in your opinion on the following matters regarding gardens.*

| 3. | In the garden would you like to have birds? Y / N Few species 1 / 2 / 3 / 4 / 5 many species |
| --- | --- |
| 4. | In the garden would you like to have flowers? Y / N Identical 1 / 2 / 3 / 4/ 5 many verities |
| 5. | In the garden would you like to have insects? Y / N Few species 1 / 2 / 3 / 4 / 5 many species |
| 6. | In the garden would you like to have flowers? Y / N Identical 1 / 2 / 3 / 4/ 5 many verities |
| 7. | In the garden would you like to have lawns (none / some / many / everywhere) |
| 8. | I feel good in garden with many different flowers?  ( strongly disagree / disagree / do not agree or disagree / agree /strongly agree ) |
| 9. | I feel good in garden with many species of birds?   ( strongly disagree / disagree / do not agree or disagree / agree /strongly agree ) |
| 10. | I feel good in garden with many species of insects?  ( strongly disagree / disagree / do not agree or disagree / agree /strongly agree ) |
| 11. | For me it is depressing to visit a garden with one type of tree?  ( strongly disagree / disagree / do not agree or disagree / agree /strongly agree ) |
| 12. | I would you like to have in one corner of this garden a natural pond?  ( strongly disagree / disagree / do not agree or disagree / agree /strongly agree ) |
| 13. | Walking on the lawns should be allowed in all gardens? Yes / No |
| 14. | Do you like to here bird song?   (not at all / sometimes / often / lots of time / at all the time) |
| 15. | Do you like to here the songs of several birds?   (not at all / sometimes / often / lots of time / at all the time) |
| 16. | Should we protect urban nature?  ( not at all / only in big parks / in all green spaces / throughout the city) |
| 17. | Should we eliminate certain species in cites?   (none / only in exceptional situations / only pest species / all insects) Which? _______________________________________________________________________________ |
| 18. | I am concerned for the disappearing of bees in Europe. (not at all / not / little / a lot / strongly) Why? ______________________________________________ |
| 19. | In Paris, should we allow the weeds to grow in the feet of trees on the sidewalks?  (I do not care / not at all / in some streets / in all streets) |

| 20. | In this garden, do you have the impression to have different type of flowers ? Yes / No  About how many ?______________ |
| --- | --- |
| 21. | In this garden, do you have the impression to have different type of insects? Yes / No  About how many? ______________ |
| 22. | In this garden, do you have the impression to have different type of birds? Yes / No  About how many? ______________ |

| 23. Gender: M / F | 24. Year of birth? ______________________ | 25. Marital status: single / couple | 2-Année de naissance? _________________ | 3-Situation familiale: seul(e) / en couple |
| --- | --- | --- | --- | --- |
|  |  |  |
| 26. Do you have children/grandchildren (below 15 years)? ____________________________________________ | | |  | |
| 27. Do you live/work nearby? Yes / No. Live: could you give us your street name___________________________  Work: where do you live? city ________________street name ___________________________  ______________________________________________________________________________________________ 28. Do you live in private house of apartment? Do you have plant in your home? Yes / No | | |  | |
| 29. Where did you pass the majority of your childhood (4-16 years)? France: _____________________________ /other: _____________________________________In: big city / average city / small agglomeration / village / farm | | |  | |
| 30. How do you judge the income of your household?   | Poor Average High | | | | | | | | | | | --- | --- | --- | --- | --- | --- | --- | --- | --- | --- | | 1 | 2 | 3 | 4 | 5 | 6 | 7 | 8 | 9 | 10 | | | |  | |
|  | | |  | |
| 31. What was the last diploma you obtain? _________________________________________________________________________________________ | | |  | |
| 32. This summer what would you like do in your vacation?_______________________________________________ ______________________________________________________________________________________________  ______________________________________________________________________________________________ | | |  | |
| 30. 33. Did you participate in our activity day at the end of last April? Yes / No | | |  | |
