# Supplementary material for: Urban Biodiversity, City-Dwellers and Conservation: How Does an Outdoor Activity Day Affect the Human-Nature Relationship?
Source: PLoS One. 2012 Jun 8;7(6):e38642. doi: 10.1371/journal.pone.0038642 (PMC3371046; doi:10.1371/journal.pone.0038642)
Supplement: Text S3 — The interview guidelines followed (set of themes and questions used to frame the interview). Face-to-face interviews were conducted, three and a half months after the activity days. (DOCX) [file pone.0038642.s003.docx]

**Interview’s questions set**

**Semi-directive interview**

**Objective : what are the consequences of the participation to the garden activity days ?**

**Introductive approach :**

To which activity day did you participate (in which garden)?

To which activities did you participate?

How long did you stay during the activity day?

With who did you participate to the animations day?

What was the motivation for your visit?

What is your opinion about the different activities?

Which interest did you find in participating to this day?

Do you see an interest in organizing similar activity days?

Did you participate to similar activities before this day? Or activities related to environment?

Did you already observe biodiversity before? Local biodiversity?In the garden?

Do you sometimes go to exhibitions, conferences… related to environment?

Did you take leaflets?

Since the activity day, did you participate to nature activities? With or without the frame of the program?

**Knowledge / Observation :**

Did you come back to the public garden? Similar observations?

Observations in the public garden or beyond?

Knowledge of birds, insects… (drawing file shown)

Do individuals demonstrate a greater knowledge of birds, insects than before?

**Perception :**

Do you have suggestions or remarks following the participation to this activity day?About the proposed activities?

What do you think about biodiversity in Paris? About conservation in general?

Does it seems interesting for you to preserve biodiversity?

Would you like these species to be preserved? (drawing file shown)

(If you have children)

Did they refer to the activity day later?

What interest do you perceive in children’s participation to the activity day?

**Action :**

Did the participation to the activity day influence your outing choices?

Which actions would you be ready to undertake to preserve biodiversity?
